# Short-term effects of endotracheal suctioning in post-cardiac arrest patients: A prospective observational cohort study

**DOI:** 10.1016/j.resplu.2022.100221

**Published:** 2022-03-19

**Authors:** Idunn Banschbach Eggen, Gunhild Brønstad, Halvor Langeland, Pål Klepstad, Trond Nordseth

**Affiliations:** a Department of Circulation and Medical Imaging, Faculty of Medicine and Heath Sciences, Norwegian University of Science and Technology, No-7491 Trondheim, Norway; b Department of Anaesthesia and Intensive Care Medicine, St. Olav’s University Hospital, No-7030 Trondheim, Norway; c Department of Anaesthesia, Molde Hospital Trust, Møre og Romsdal Health Authority, No-6412 Molde, Norway

**Keywords:** Post-cardiac arrest syndrome, Endotracheal suctioning, Circulatory failure, Swan-Ganz, Invasive ventilation, OHCA, Out-of-hospital cardiac arrest, ROSC, Return of spontaneous circulation, ETS, Endotracheal suctioning, SOFA, Sequential Organ Failure Assessment, RASS, Richmond Agitation-Sedation Scale, MAAS, Motor Activity Assessment Scale, ICU, Intensive care unit, PAC, Pulmonary artery catheter, PEA, Pulseless electrical activity

## Abstract

**Background:**

Endotracheal suctioning (ETS) is required in critically ill patients but may lead to adverse physiologic effects. The aim of this study was to investigate risk factors associated with adverse respiratory and circulatory effects of ETS, in post-cardiac arrest patients receiving controlled ventilation.

**Methods:**

Patients with return of spontaneous circulation after out-of-hospital cardiac arrest were followed the first five days in the intensive care unit (ICU). For each ETS procedure performed, data were extracted from the electronic ICU records 10 min before and until 30 min after the procedure. Adverse events were defined as heart rate > 120 beats/min, systolic blood pressure > 200 or < 80 mmHg or SpO_2_ < 85%. Multivariate logistic regression was applied with SpO_2_ < 85% and systolic blood pressure < 80 mmHg as primary outcomes.

**Results:**

For the 36 patients included in the study, the median number of ETS-procedures per patient was 13 (range 1–33). Oxygen desaturation occurred in 10.3% of procedures and severe hypotension in 6.6% of procedures. In the multivariate analysis, dose of noradrenaline, light sedation and oxygen desaturation prior to suctioning were associated with increased risk of oxygen desaturation. Doses of noradrenaline, suction with manual ventilation, suction in combination with patient repositioning, and first day of treatment in the ICU were significantly associated with severe hypotension.

**Conclusions:**

The risk of circulatory and respiratory deterioration during ETS in post-cardiac arrest patients is increased the first day of ICU care, and related to sedation, dose of noradrenaline and pre-procedure hypoxemia.

## Introduction

In intubated patients treated in an intensive care unit (ICU), endotracheal suctioning (ETS) mechanically removes accumulated pulmonary secretions. ETS secures a patent airway and reduces the risk of tracheal tube obstruction, as well as the risk of lung consolidation and atelectasis that may result in inadequate ventilation of the lung.^1^^.^ However, there are several complications associated with ETS, such as cardiovascular instability, hemorrhagic secretions, bronchospasm and atelectasis.2., 3., 4.^.^ ETS is shown to increase heart rate and mean arterial pressure, and to increase the risk for hypoxemia.4., 5., 6., 7., 8.^.^ The American Association of Respiratory Care recommends that ETS should only be performed when clinically indicated.^2^^.^ Thus, the frequency with which ETS is performed will differ between patients and has been reported to vary between 8 and 17 times per day in previous studies.^9^^.^

Most studies on ETS have been performed on mixed ICU populations and some have excluded patients with vasopressor infusion.8., 10., 11., 12.^.^ There is a lack of studies investigating the effects of ETS in patients that may be more vulnerable for adverse circulatory and respiratory events, such as patients with return of spontaneous circulation (ROSC) after out-of-hospital cardiac arrest (OHCA). These patients have experienced peri-arrest myocardial ischemia and may have post-arrest myocardial dysfunction, which may increase the risk of circulatory disturbances when receiving ETS. The aim of this study was to assess the incidence and risk factors for adverse circulatory and respiratory events following ETS, in the early phase of ICU treatment after OHCA.

## Methods

### Study design and setting

This is a planned sub-study of a prospective observational study of 50 patients admitted to St. Olav’s University Hospital after OHCA between January 2016 and November 2017.^13^^.^ This is a tertiary care hospital in Trondheim, Norway, with a catchment population of approximately 700 000. The study was approved by the Regional Committee for Medical and Health Research Ethics, Central Norway Health Region (REK Midt, No. 2015/1807). Written informed consent was obtained in all cases from either the patient, or the next-of-kin if the patient was unable to consent.

### Eligibility

All comatose patients, requiring mechanical ventilation, admitted to an ICU with ROSC after OHCA were included in the analysis. The following patients were excluded from the study^13^: pregnant patients, patients less than 18 years of age, assumed septic or anaphylactic etiology of cardiac arrest, patients transferred from another hospital, decision to limit life-sustaining therapy upon arrival, acute cardiothoracic surgery or a need for mechanical circulatory support.

### Study period

The patients were followed for five consecutive days or until death or extubation. The five-day follow-up in this sub-study was a consequence of this being the time frame for collection and analysis of biomarkers in the main study. All ETS procedures were registered during the study period using the electronic critical care management system (Picis CareSuite, Optum Inc., USA). For all ETS procedures, the time of start and end of each procedure was registered manually in the electronic critical care management system by the nurses performing the procedure. ICU day zero was defined from time of arrival in the ICU until 6 AM the following morning. The following ICU days were defined from 6 AM until 6 AM.

### Study procedure

#### Circulatory and respiratory measurements

All patients received an intra-arterial cannula for invasive blood pressure measurements. Patients without contraindications received a pulmonary artery catheter (PAC) (Swan-Ganz CCOmbo, Edwards Lifescience, USA). The following data were recorded by the electronic critical care management system: heart rate (HR), systolic blood pressure (SBP); central venous pressure (CVP); pulmonary artery pressure; systemic vascular resistance (SVR); mixed venous oxygen saturation (SvO_2_); cardiac output (CO); peripheral transcutaneous oxygen saturation (SpO_2_); respiratory rate; fraction of inspired oxygen (FiO_2_); minute ventilation (MV); positive end-expiratory pressure (PEEP); setting of pressure control over PEEP (SetPC) and dose of noradrenaline (norepinephrine, μg/kg/min) and other medications. Data were recorded once every minute during the observation period. A more detailed description of study protocol, data collected, and the post-cardiac arrest care given has been published previously.13., 14.^.^

#### Endotracheal suction procedures

ETS was performed with a 10–14 French suction catheter, depending on endotracheal tube size, either via a closed or open suction system, with a maximum negative pressure of −150 mmHg, to secure patent airways when indicated according to local guidelines. Indications for ETS included: sounds from the respiratory tract indicating sputum retention, visual sputum in the tube, suspected aspiration of gastric content, reduced ability to generate an effective cough, increased peak inspiratory pressure during volume-controlled mechanical ventilation, oxygen desaturation and/or deteriorating blood gas values. Hypoxemic patients were pre-oxygenated with 100% oxygen for 1 min before the procedure. If significant oxygen desaturation occurred after the procedure, post-oxygenation was provided. If clinically indicated, the procedure was performed in combination with patient repositioning and defined as a ‘combined procedure’. If ventilation was primarily given by a ventilation bag during the procedure, the procedure was labelled as ‘manual ventilation’ (with or without combination with patient repositioning). According to local guidelines, most ETS procedures were performed with a shallow suctioning technique. Deep suctioning was performed if clinically indicated, but depth of suctioning was not registered in this study.

#### Registration of endotracheal suction and concomitant events

The time of start and end of each ETS procedure was assessed based on registrations in the electronic records. The time of other clinical events, such as position changes, were also registered. In the ICU, the following scoring systems were used to evaluate clinical conditions and level of sedation: daily Clinical Pulmonary Infection Score (CPIS)^15^; daily Sequential Organ Failure Assessment (SOFA) score^16^^.^ Richmond Agitation-Sedation Scale (RASS)^17^^.^ or Motor Activity Assessment Scale (MAAS).^18^ As the use of the MAAS was discontinued in April 2017, the reported MAAS value was converted into the corresponding RASS value.

ETS procedures were excluded from the analysis when combined with other interventions within 10 min prior to and until 10 min after ETS, such as bronchoscopy, laryngoscopy, change of tracheal tube, extubation, placement of a nasogastric tube or cardioversion. The procedure was also excluded if ETS was performed within 30 min prior to the following ETS or if there were technical issues related to data collection (e.g. data not registered every minute). If patient repositioning was done simultaneously with ETS, the registered event was labeled ‘combined procedure’. If change of patient position occurred after 10 min from start of the procedure, data was not collected after start of the reposition.

### Statistics

In the statistical analysis, each included ETS procedure was treated as an independent event. Each event was defined as 10 min before initiation of ETS until 30 min after start of the procedure. To correct for possible erroneous registrations, values outside a clinically probable range (Supplementary Table 1) were treated as missing data. No formal sample size calculation was performed.

Circulatory and respiratory effects of ETS were defined if the effects occurred within 15 min after the initiation of the procedure. The timeframe was selected to include both immediate effects of ETS, as well as more delayed effects of the procedure. Adverse effects occurring later than 15 min after initiation of ETS were considered less likely to be related to the procedure. Changes in variables are given as percentage change from pre-procedural measurements (“baseline”), the latter was defined as the mean value between 10 and 5 min before ETS was performed. This period was chosen to avoid that baseline estimates were affected by any inaccuracies in the manually recorded times for start of ETS. A circulatory effect after initiation of ETS was defined as either tachycardia (HR > 120 beats per minute), severe hypertension (SBP > 200 mm Hg), or severe hypotension (SBP < 80 mmHg). A respiratory effect was defined if SpO_2_ fell below 85% after initiation of ETS.

Univariate logistic regression was applied to identify procedure-specific risk factors, with SPB < 80 mmHg and SpO_2_ < 85% as outcome variables. The following procedure-specific covariates were included in the regression analysis: ICU Day 0–5, dose of noradrenaline five minutes prior to the procedure, SOFA score, CPIS score, RASS score, suction with manual ventilation, combined suction and patient repositioning, frequency of suctioning, FiO_2_ level and baseline oxygen saturation less than 92%. Covariates with *p* < 0.20 in the univariate analysis, were included in the multivariate logistics regression model. A p-value < 0.05 was considered statistically significant and 95% confidence intervals (95% CI) were reported for the estimated odds ratios (OR). Concordance statistic (C-statistic) was applied to determine the predictive ability of the models. Data were analyzed with the software Matlab version 2020a (MathWorks, Natick, MA, USA). Statistical analyses were performed using R version 4.0.3 and RStudio, applying the packages ‘ggplot2′, ‘dplyr’, ‘tidyr’, ‘readr’ and ‘DescTools’.19., 20.^.^

## Results

### Demographics

Of 50 patients included in the main study, nine patients did not receive mechanical ventilation, three had no ETS procedures performed, and two patients were excluded because of lack of data on ETS procedures. Thirty-six patients were included in the final analysis. Details of patient inclusion is presented in Supplementary Fig. 1 and patient characteristics are presented in Table 1. The median age was 67.5 (range 33–90) years, 86% were male and 78% had an initial rhythm of ventricular fibrillaton. The median time to ROSC was 25.5 (IQR 14) minutes. Twenty-six of the patients received a PAC. Fifteen patients ended the observational period before day five, reasons included death (n = 8), transfer to a general ward (n = 6) and acute cardiothoracic surgery (n = 1).

**Table 1:** Patient characteristics.

Patients observed, n	36	
Male, n (%)	31	(86)
Age (y), median (IQR)	67.5	(16.7)
BMI (kg/m^2^), median (IQR)	26.7	(6)
Charlson Comorbidity Index, median (IQR)	3.5	(2.3)
Location of cardiac arrest, n (%)		
Home	12	(33)
Public	15	(42)
Other	9	(25)
Witnessed cardiac arrest, n (%)	30	(83)
Bystander CPR, n (%)	32	(89)
Time to basic life support (minutes), median (IQR)	1	(1)
Time to defibrillation (minutes), median (IQR)	11	(9)
Time to ROSC (minutes), median (IQR)	25.5	(14)
Cause of cardiac arrest, n (%):		
Cardiac	32	(89)
Asphyxia	3	(8)
Other	1	(3)
Initial monitored rhythm, n (%)		
Asystole	1	(3)
Ventricular fibrillation	28	(78)
PEA	6	(17)
Other	1	(3)
Certain pulmonary aspiration, n (%)	9	(25)
Shock, n (%)	14	(39)
SAPS II, median (IQR)	67.5	(16.2)
Number of suctions per patient, median (range)	13	(1–33)

Demographic and clinical data for patients included. IQR = interquartile range; BMI = body mass index; CPR = cardiopulmonary resuscitation; ROSC = return of spontaneous circulation; PEA = pulseless electrical activity; SAPS II = Simplified Acute Physiology Score II.

During the follow-up time, each patient contributed with a median observation time of 112.5 (range 11.4–142.6) hours and had a median of 13 (range 1–33) suction procedures included in the analysis. Of the 600 ETS procedures registered, 163 procedures were excluded for the following reasons: inaccurate recordings (n = 89), failure to collect data with sufficient resolution (n = 37), ETS procedures performed at short intervals (n = 28), or other major clinical interventions (n = 9). A total of 437 procedures were included in the final analysis and the clinical characteristics are demonstrated in Table 2. Among the included procedures, a total of 171 included patient repositioning and were classified as ‘combined procedures’. In 56 procedures, ventilation was given by ventilation bag alone, of which 25 of these were ‘combined procedures’.

**Table 2:** Procedure characteristics (n = 437).

Length of procedure in minutes*, median (IQR)	2	(2)
ICU day 0†, no. (%)	19	(4)
ICU day 1, no. (%)	58	(13)
ICU day 2, no. (%)	101	(23)
ICU day 3, no. (%)	108	(25)
ICU day 4, no. (%)	93	(21)
ICU day 5, no. (%)	58	(13)
SOFA score total, median (IQR)	11	(3)
SOFA score respiration, median (IQR)	3	(1)
SOFA score cardiovascular, median (IQR)	3	(3)
Degree of sedation*:		
Not sedated (RASS ≥ 0), no. (%)	9	(2)
Light sedation (RASS −2 & −1), no. (%)	17	(4)
Moderate sedation (RASS −3), no. (%)	115	(26)
Deep sedation (RASS −5 & −4), no. (%)	292	(67)
CPIS score, median (IQR)	6	(3)
Noradrenaline dose‡ (μg/kg/min), median (IQR)	0.03	(0.08)
Baseline oxygen desaturation††, no. (%)	41	(9)
Number of suctions per patient per day, median (range)	6	(1–14)
More than 6 suctions per day, no. (%)	179	(41)
FiO_2_‡‡ >= 0.6, no. (%)	13	(3)
PEEP‡‡ (cm H_2_O), median (IQR)	8.2	(2.2)
SetPC‡‡ (cm H_2_O), median (IQR)	14	(4)
Suction with manual ventilation, no. (%)	56	(13)
Combined procedure, no. (%)	171	(39)
Received infusion of muscle relaxant (cisatracurium)	18	(4)

Clinical characteristics of the endotracheal suctioning procedures performed. IQR = interquartile range; SOFA = Sequential Organ Failure Assessment score; RASS = Richmond Agitation-Sedation Scale; CPIS = Clinical Pulmonary Infection Score; FiO_2_ = fraction of inspired oxygen; PEEP = positive end-expiratory pressure; SetPC = Pressure control value above PEEP on ventilator.

* The proportion of missing measurements for procedural length was 11.7% and 1% for RASS-score.

† The median length of first ICU day was 13 (range 1–20) hours.

†† Mean value of SpO_2_ < 92% during t = −10 to t = −5.

‡ Values measured at t = −5.

‡‡ Values measured at t = −10.

### Clinical changes after the suction procedures

**Fig. 1:**
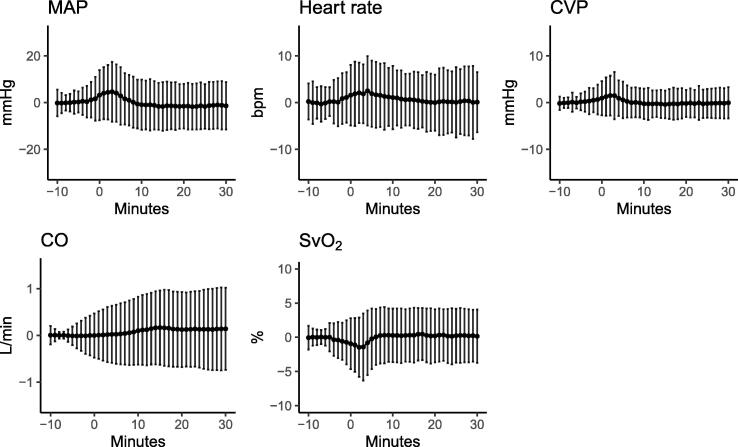
Circulatory effects of endotracheal suctioning. Mean values shown as percentage change from the baseline values of circulatory variables. The bars represent the standard deviations (±SD) from the mean and minutes = 0 marks the initiation of the endotracheal suctioning procedure. Baseline value is the mean value between minutes = −10 to minutes = −5. MAP: mean arterial pressure; CVP: central venous blood pressure; CO: cardiac output; SvO_2_: mixed venous oxygen saturation.

**Fig. 2:**
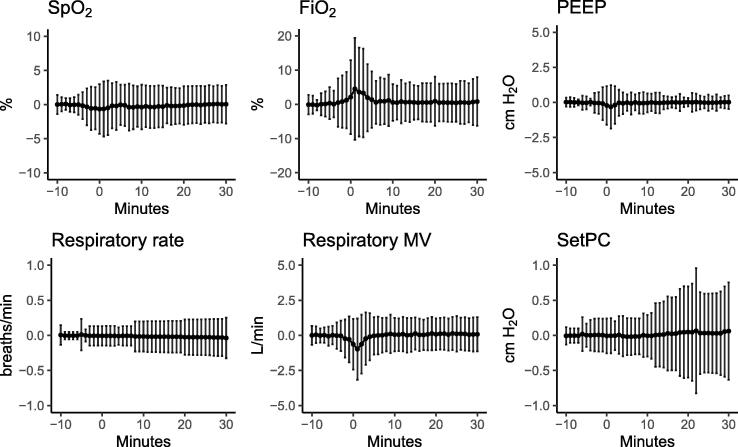
Respiratory effects of endotracheal suctioning Mean values shown as percentage change from the baseline values of respiratory variables. The bars represent the standard deviations (±SD) from the mean and minutes = 0 marks the initiation of the endotracheal suctioning procedure. Baseline value is the mean value during minutes = −10 to minutes = −5. SpO_2_: peripheral transcutaneous oxygen saturation; FiO_2_: fraction of inspired oxygen; PEEP: positive end-expiratory pressure; MV: minute ventilation; SetPC: setting of pressure control above PEEP.

**Fig. 3:**
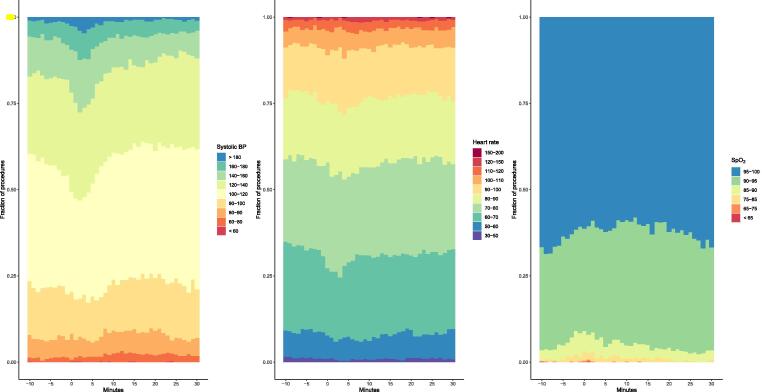
Effects of endotracheal suctioning on systolic blood pressure, heart rate and transcutaneous oxygen saturation Stacked bar plots showing all measured values, zero on the x-axis marks the initiation of the endotracheal suctioning procedure. BP: blood pressure. SpO_2_: peripheral transcutaneous oxygen saturation.

During the study period, respiratory and/or circulatory deteriorations were observed in 90 (20.6%) procedures performed in 28 (77.8%) patients. Respiratory deterioration occurred in 10.3% of the procedures in 69.4% of patients, whereas circulatory deterioration occurred in 13.3% of the procedures in 61.1% of patients. Severe hypotension was the most common adverse effect, occurring in 6.6% of procedures in 41.7% of the patients. The detailed clinical effects of ETS on circulatory and respiratory variables are demonstrated in Fig. 1, Fig. 2, Fig. 3. The circulatory and respiratory changes were most pronounced during the first 5 min after the start of ETS, with most values returning to baseline within 10 min.

### Risk factors for clinical deterioration

The results from the logistic regression analyses are shown in Table 3. In the multivariate model, insufficient sedation (OR 4.22), hypoxemia (SpO_2_ < 92%) prior to suctioning (OR 3.24) and dose of noradrenaline (OR 1.07 per one unit increase in μg/kg/min) were significant risk factors for oxygen desaturation. The model’s discriminative ability was good (C-statistic 0.71). First ICU day (OR 5.53), suction with manual ventilation (OR 3.56), “combined procedure” (OR 2.73) and dose of noradrenaline (OR 1.10) were significant risk factors for severe hypotension. The discriminative ability of the model was strong (C-statistic 0.81).

**Table 3:** Logistic regression analysis. Complications of endotracheal suctioning.

	Univariate analysis			Multivariate analysis		
	OR	95% CI	*p*	OR	95% CI	*p*
*Oxygen desaturation (SpO_2_ < 85%)*						
ICU day 0†	4.49	1.51–12.05	0.004	3.11	0.76–11.02	0.091
ICU day 1	1.75	0.75–3.72	0.165	0.82	0.28–2.19	0.701
ICU day 2	0.82	0.36–1.69	0.602			
ICU day 3	0.63	0.27–1.34	0.258			
ICU day 4	0.78	0.33–1.66	0.545			
ICU day 5	0.80	0.27–1.95	0.652			
SOFA score total	1.18	1.03–1.36	0.018			
SOFA score respiration	1.83	1.17–2.96	0.011			
SOFA score cardiovascular	1.73	1.28–2.50	0.001			
CPIS score	0.97	0.84–1.12	0.636			
Light sedation (RASS −2 – 6)	2.31	0.74–6.06	0.111	4.22	1.24–12.58	**0.013**
Moderate sedation (RASS −3)	0.60	0.25–1.28	0.217			
Deep sedation (RASS −5 – −4)	1.13	0.58–2.30	0.731			
Suction with manual ventilation	1.84	0.79–3.91	0.133	2.11	0.86–4.81	0.085
Combined procedure	1.90	1.02–3.57	0.042	2.07	1.05–4.15	0.037
More than 6 suctions per day	0.49	0.24–0.95	0.043	0.65	0.28–1.45	0.299
FiO_2_‡ >= 0.6	4.23	1.11–13.65	0.021	1.07	0.17–4.95	0.940
Baseline oxygen desaturation††	4.01	1.78–8.56	<0.001	3.24	1.25–7.86	**0.012**
Noradrenaline dose‡‡	1.08	1.04–1.12	<0.001	1.07	1.02–1.12	**0.006**
*Hypotension (Systolic blood pressure < 80 mmHg)*						
ICU day 0†	7.93	2.59–22.11	<0.001	5.53	1.30–20.38	**0.013**
ICU day 1	1.40	0.45–3.54	0.516			
ICU day 2	0.86	0.31–2.05	0.749			
ICU day 3	0.47	0.14–1.24	0.167	0.81	0.22–2.35	0.713
ICU day 4	0.96	0.35–2.30	0.936			
ICU day 5	0.47	0.07–1.61	0.306			
SOFA score total	1.30	1.09–1.57	0.005			
SOFA score respiration	2.12	1.21–3.87	0.012			
SOFA score cardiovascular	2.06	1.35–3.61	0.004			
CPIS score	1.14	0.95–1.38	0.177	1.08	0.88–1.35	0.479
Light sedation (RASS −2 – 6)	0.56	0.03–2.82	0.580			
Moderate sedation (RASS −3)	1.11	0.45–2.52	0.803			
Deep sedation (RASS −5 – −4)	1.02	0.46–2.43	0.961			
Suction with manual ventilation	2.86	1.14–6.59	0.018	3.56	1.33–8.98	**0.008**
Combined procedure	2.34	1.09–5.14	0.030	2.73	1.19–6.58	**0.020**
More than 6 suctions per day	0.63	0.27–1.38	0.264			
FiO_2_‡ >= 0.6	7.04	1.81–23.31	0.002	1.80	0.26–9.46	0.517
Baseline oxygen desaturation††	2.14	0.69–5.55	0.144	1.06	0.26–3.36	0.924
Noradrenaline dose‡‡	1.11	1.06–1.16	<0.001	1.10	1.04–1.15	**<0.001**

Univariate and multivariate logistic regression analysis of complications of endotracheal suctioning. ICU = intensive care unit; SOFA = Sequential Organ Failure Assessment score; CPIS = Clinical Pulmonary Infection Score; RASS = Richmond Agitation-Sedation Scale; FiO_2_ = fraction of inspired oxygen; SpO_2_ = peripheral transcutaneous oxygen saturation.

† The median length of first ICU day was 13 (range 1–20) hours.

†† Mean value of SpO_2_ < 92% during t = −10 to t = −5.

‡ Values measured at t = −10.

‡‡ Dose measured at t = −5, effect estimates represent increase of 0,01 µg/kg/min of noradrenaline.

## Discussion

We observed that ETS in critically ill patients after cardiac arrest was associated with increased risk of circulatory and respiratory deteriorations, which were of short durations. Severe hypotension or desaturation occurred in approximately 6% and 10% of the procedures, respectively, which may be harmful for post cardiac arrest patients who have experience peri-and post arrest ischemia of the brain and myocardium. Higher dose of noradrenaline, insufficient sedation and hypoxemia prior to suctioning were independent risk factors for desaturation. With respect to the risk of severe hypotension during the procedure, higher dose of noradrenaline, suction with manual ventilation, combined procedures (i.e. suction and patient repositioning) and procedure performed on the first day in ICU were found to be significant risk factors in the multivariate model. We believe that the effect estimates provided, on how much each risk factor contributes to the risk of adverse events, represent important and novel findings in patients with OHCA treated in an ICU.

Compared to previously published studies, we found higher incidences of oxygen desaturation and severe hypotension.4., 21.^.^ Differences in the definition of adverse effects, suctioning techniques, use of absolute versus relative cutoff values, types of patients included, and study size might explain the differences. Although, circulatory variables in general returned to baseline values within 10 min, changes in heart rate and MAP were observed for longer periods than previously reported.6., 8.^.^ Changes in ventilatory settings were small to moderate before and after procedures, as judged by Fig. 2.

The decrease in peripheral oxygen saturation before the ETS might be explained by prior events leading to desaturation, as well as patient overall physiologic status reflected in the SOFA score and FiO_2_. The latter two variables were univariately associated with oxygen desaturation in the logistic regression model. Pre-procedural hypoxemia is a possible source for confounding by indication, as this may represent an indication for why ETS was initiated in the first place. Another factor that may modify the degree of oxygen desaturation during ETS is that for some patients the FiO_2_ was increased either before or after the ETS, which influence the frequencies of observed oxygen desaturations.

The dose of noradrenaline was independently associated with both oxygen desaturation and severe hypotension, suggesting a connection between adverse effects of ETS and increased circulatory instability. In our study, the first ICU Day was the strongest independent predictor for severe hypotension during ETS, which may be related to haemodynamic instability being more pronounced in the first hours after OHCA. Laurent and co-workers^22^^.^ found that severe myocardial dysfunction may occur independently of underlying coronary disease in post-cardiac arrest patients, and that the haemodynamic changes may be explained with myocardial stunning and increased vasodilatation. However, only 4% of ETS procedures were done during the first day in ICU. ETS were most frequently performed between ICU days two and four, which can be explained by an increased necessity for ETS with prolonged intubation. It also reflects variable duration of the first day in our study due to different time of inclusion (range 13–24 h), as well as reluctancy to perform ETS in patients who were circulatory unstable. Severe circulatory failure necessitating higher doses of noradrenaline may also cause hypoperfusion, which may impact on SpO2 measurements.^23^^.^

Combined suction and patient repositioning were shown to be associated with severe hypotension, which might indicate that these types of procedures represent a greater strain on the patient. Some aspects of the ETS procedure, such as suctioning depth and the use of an open or closed suctioning system, were not assessed in the analysis. Depth of insertion of the suctioning catheter may have impacted results, as shallow ETS has been associated with fewer adverse effects when compared to deep ETS.^21^^.^

Insufficient sedation was the strongest independent predictor for oxygen desaturation. In a study on physiologic impacts of closed ETS on spontaneously breathing patients on mechanical ventilation,^8^ Seymour and co-workers found that the patients had more pronounced changes in physiological variables compared to earlier studies, with deeper sedated patients. They suggested that deep sedation depresses both laryngeal and tracheal reflexes, and blunt the physiologic effect of airway manipulation. This may explain why patients that were lighter sedated were more vulnerable to changes in physiological variables during ETS. Baseline oxygen saturation (SpO_2_ < 92%) was also a strong independent predictor for oxygen desaturation after ETS. This is expected, as it is likely that patients that have a lower saturation prior to the procedure will be more vulnerable to changes in oxygen consumption and -delivery.

## Limitations

This was a single center study with a low number of patients included. Firstly, some patients had longer ventilator time, or had ETS done more frequently, and thus contributed with a higher number of observations. This may introduce statistical dependence between procedures. Secondly, the study contains possible confounding factors that might have impacted the results, such as hypoxemia prior to the procedures and hypoperfusion due to circulatory instability and the use of noradrenaline. Thirdly, the analysis only investigated the short-time effect of ETS and cannot be used to assess the long-term effects of the procedure in these patients. Fourthly, as we applied logistic regression on events that occurred several times in the same patients, we only assessed procedure-specific risk factors associated with deterioration and not patient-specific risk factors. In addition, we assumed the procedures to be independent events in the analysis. We included 19 covariates in the regression analysis, which may have increased the risk of “overfitting” and thus overestimating the effects of individual covariates (increasing the risk of type 1 errors). Fifthly, a comparison with ICU patients admitted for other reasons could have strengthened the assessment of whether post-cardiac arrest patients are more vulnerable to ETS than other ICU patients. Lastly, ETS is a manually performed procedure. Our study design therefore relies on compliance by the nurse for both correct execution of ETS, as well as correct timing and recording of the procedures. As the main study was not specifically designed to assess ETS, the clinical documentation of procedural length in the ICU records may be imprecise and include preparations, re-positioning of the patient and other elements.

## Conclusions

We found that ETS in patients with OHCA, treated in an ICU, resulted in severe hypotension or oxygen desaturation in 20% of the ETS procedures in 78% of the patients. Higher dose of noradrenaline, insufficient sedation and hypoxemia prior to suctioning were risk factors independently associated with increased risk of oxygen desaturation. These results may identify risk factors that should be considered, and if possible corrected, before performing ETS in ICU patients after OHCA.

## Conflicts of Interest

The authors have no conflicts of interest to report.

## CRediT authorship contribution statement

**Idunn Banschbach Eggen:** Conceptualization, Methodology, Software, Investigation, Writing – original draft, Writing – review & editing, Visualization. **Gunhild Brønstad:** Conceptualization, Methodology, Software, Investigation, Writing – original draft, Writing – review & editing, Visualization. **Halvor Langeland:** Conceptualization, Methodology, Writing – review & editing, Supervision, Project administration. **Pål Klepstad:** Conceptualization, Methodology, Investigation, Resources, Writing – review & editing, Supervision, Project administration, Formal analysis. **Trond Nordseth:** Methodology, Data curation, Software, Investigation, Data curation, Writing – original draft, Writing – review & editing, Visualization, Supervision, Formal analysis.

## Appendix A Supplementary material

Supplementary data 1 Flowchart summarizing patient enrollment. ICU: intensive care unit; OHCA: out-of-hospital cardiac arrest; ROSC: return of spontaneous circulation; CA: cardiac arrest.

Supplementary data 2 Minimum and maximum cut-off values for the physiological variables used for filtration of data for erroneous registrations. Bpm: beats per minute; BP: blood pressure; SpO_2_: peripheral transcutaneous oxygen saturation; FiO_2_: fraction of inspired oxygen; PEEP: positive end-expiratory pressure; SetPC: setting of pressure control above PEEP; MV: minute ventilation.
