# Supplementary material for: Short-term effects of endotracheal suctioning in post-cardiac arrest patients: A prospective observational cohort study
Source: Resusc Plus. 2022 Mar 19;10:100221. doi: 10.1016/j.resplu.2022.100221 (PMC8938328; doi:10.1016/j.resplu.2022.100221)
Supplement: Supplementary data 1 — Flowchart summarizing patient enrollment. ICU: intensive care unit; OHCA: out-of-hospital cardiac arrest; ROSC: return of spontaneous circulation; CA: cardiac arrest. [file mmc1.pdf]

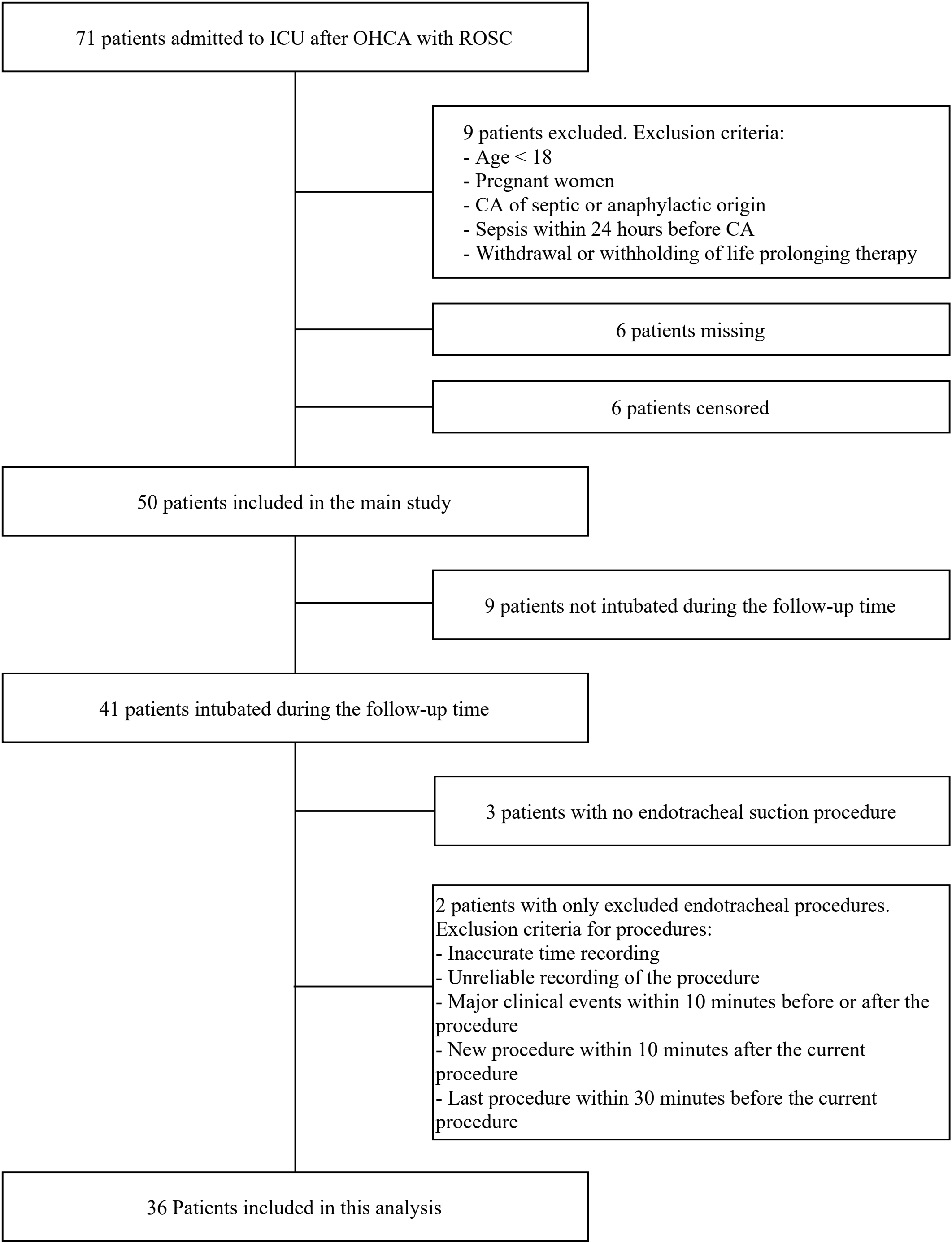
71 patients admitted to ICU after OHCA with ROSC
9 patients excluded. Exclusion criteria:
- Age < 18
- Pregnant women
- CA of septic or anaphylactic origin
- Sepsis within 24 hours before CA
- Withdrawal or withholding of life prolonging therapy
6 patients missing
6 patients censored
50 patients included in the main study
9 patients not intubated during the follow-up time
41 patients intubated during the follow-up time
3 patients with no endotracheal suction procedure
2 patients with only excluded endotracheal procedures.
Exclusion criteria for procedures:
- Inaccurate time recording
- Unreliable recording of the procedure
- Major clinical events within 10 minutes before or after the procedure
- New procedure within 10 minutes after the current procedure
- Last procedure within 30 minutes before the current procedure
36 Patients included in this analysis
