# Supplementary material for: Short-term effects of endotracheal suctioning in post-cardiac arrest patients: A prospective observational cohort study
Source: Resusc Plus. 2022 Mar 19;10:100221. doi: 10.1016/j.resplu.2022.100221 (PMC8938328; doi:10.1016/j.resplu.2022.100221)
Supplement: Supplementary data 2 — Minimum and maximum cut-off values for the physiological variables used for filtration of data for erroneous registrations. Bpm: beats per minute; BP: blood pressure; SpO2: peripheral transcutaneous oxygen saturation; FiO2: fraction of inspired oxygen; PEEP: positive end-expiratory pressure; SetPC: setting of pressure control above PEEP; MV: minute ventilation. [file mmc2.docx]

| Supplementary Table 1. Minimum and maximum cut-off values | | | | |
| --- | --- | --- | --- | --- |
| Variable | |  | Min | Max |
| Heart rate (bpm) | |  | 30 | 250 |
| Systolic BP (mmHg) | |  | 40 | 250 |
| Diastolic BP (mmHg) | |  | 20 | 150 |
| Mean Arterial Pressure (mmHg) | |  | 20 | 150 |
| Central Venous Pressure (mmHg) | |  | 0 | 40 |
| SpO_2_ (%) | |  | 55 | 100 |
| FiO_2_ (%) | |  | 21 | 100 |
| PEEP (cm H_2_O) | |  | 0 | 20 |
| SetPC (cm H_2_O) | |  | 6 | 40 |
| Respiratory rate (breaths/min) | | | 5 | 40 |
| Respiratory MV (L/min) | |  | 0.3 | 15 |
|  |  | |  |  |
| bpm = Beats per minute  BP = Blood pressure  SpO_2_ = Peripheral transcutaneous oxygen saturation  FiO_2_ = Fraction of inspired oxygen  PEEP = Positive End-Expiratory Pressure  SetPC = Setting of Pressure Control over PEEP  MV = Minute volume | |  |  |  |
